# Electrochemical Polymerization of PEDOT–Graphene Oxide–Heparin Composite Coating for Anti-Fouling and Anti-Clotting of Cardiovascular Stents

**DOI:** 10.3390/polym11091520

**Published:** 2019-09-18

**Authors:** Ming-Chien Yang, Hui-Ming Tsou, Yu-Sheng Hsiao, Yu-Wei Cheng, Che-Chun Liu, Li-Ying Huang, Xin-Yao Peng, Ting-Yu Liu, Ming-Chi Yung, Chuan-Chih Hsu

**Affiliations:** 1 Department of Materials Science and Engineering, National Taiwan University of Science and Technology, Taipei 10607, Taiwan; 2 Department of Materials Engineering, Ming Chi University of Technology, New Taipei City 24301, Taiwan; 3 Division of Cardiovascular Surgery, Department of Surgery, School of Medicine, Taipei Medical University Hospital, Taipei 11031, Taiwan

**Keywords:** PEDOT, electrochemical polymerization, SUS316L, biocompatibility, anti-fouling, anti-clotting

## Abstract

In this study, a novel hemocompatible coating on stainless steel substrates was prepared by electrochemically copolymerizing 3,4-ethylenedioxythiophene (EDOT) with graphene oxide (GO), polystyrene sulfonate (PSS), or heparin (HEP) on SUS316L stainless steel, producing an anti-fouling (anti-protein adsorption and anti-platelet adhesion) surface to avoid the restenosis of blood vessels. The negative charges of GO, PSS, and HEP repel negatively charged proteins and platelets to achieve anti-fouling and anti-clotting. The results show that the anti-fouling capability of the poly(3,4-ethylenedioxythiophene) (PEDOT)/PSS coating is similar to that of the PEDOT/HEP coating. The anti-fouling capability of PEDOT/GO is higher than those of PEDOT/HEP and PEDOT/PSS. The reason for this is that GO exhibits negatively charged functional groups (COO^−^). The highest anti-fouling capability was found with the PEDOT/GO/HEP coating, indicating that electrochemical copolymerization of PEDOT with GO and HEP enhances the anti-fouling capability. Furthermore, the biocompatibility of the PEDOT coatings was tested with 3T3 cells for 1–5 days. The results show that all PEDOT composite coatings exhibited biocompatibility. The blood clotting time (APTT) of PEDOT/GO/HEP was prolonged to 225 s, much longer than the 40 s of pristine SUS316L stainless steel (the control), thus greatly improving the anti-blood-clotting capability of cardiovascular stents.

## 1. Introduction

Cardiovascular disease has evolved as a health problem and the main cause of death [1,2,3]. Coronary artery disease (CAD) is the most common disease in the modern world, causing about 2 in every 10 deaths [4]. Since the 1980s, stent implantation has become an important therapeutic strategy for narrowing of the aorta [5]. A coronary stent was approved by the FDA in 1993 [6]. The materials for cardiovascular stents are usually 316L stainless steel, tantalum, and CoCr alloys [7]. 316L stainless steel exhibits corrosion resistance and has been proven to be biocompatible with humans [8,9]. Nowadays there are many different stents, including cardiovascular stents, drug-eluting stents, and biodegradable stents [10,11]. Biodegradable stents have the lowest tensile strength [12]; other limitations of biodegradable stents include inability of balloon expansion [13] and a stenosis rate similar to that of traditional coronary stents [14]. In general, the restenosis rate of unmodified cardiovascular stents is about 25% [15].

Graphene oxide (GO) possesses oxygen functional groups on the basal plane and edge. In graphene oxide, the C/O ratio is about 4:1 to 2:1 [16,17,18]. In addition, due to the oxygen functional groups, the thickness of GO is about 1 nm thicker than the theoretical value (0.34 nm) [19]. Furthermore, the negative charges make GO easy to combine with positively charged monomers. This property can be applied to layer-by-layer techniques for biosensors [20,21,22]. Recent reports have suggested that GO is suitable for the growth of cells [23,24]. On the other hand, the oxygen functional groups on the GO surface are well dispersed in water and can also be used as a combining site for GO-based composites. Thus, GO was combined with conducting polymer to form conducting composite films, thereby improving its composite conductivity [25,26].

A series of polymeric materials have been widely applied as anti-fouling coatings due to their excellent characteristics, such as low elastic modulus, low surface free energy, and low surface roughness [27,28,29,30,31]. Polystyrene sulfonate (PSS), a highly conductive polymer, is commonly used for solar films. In addition, PSS is also useful for the treatment of hyperkalemia [32]. Polystyrene sulfonate is hydrophobic and needs to be doped with 3,4-ethylenedioxythiophene (EDOT) to increase its solubility. According to reports, a common ratio of 1:2.5 is the best [33].

Heparin (HEP) played a crucial role in the development of treatment for thrombosis [34]. When human tissue is damaged, the coagulation mechanism is activated. Thrombin transforms fibrinogen into fibrin monomers, and these monomers eventually transform into fibrin threads. The fibrin threads form a network to trap platelets, blood cells, and plasma-related coagulants, resulting in blood clot formation [35,36,37]. Positively charged EDOT monomers and negatively charged, anticoagulated heparin can be adopted to conduct copolymerization and synthesize poly(3,4-ethylenedioxythiophene) (PEDOT)/HEP films.

In this study, the restenosis rate was reduced by using electrochemical polymerization on the surface of an SUS316L stent. This coating was composed of EDOT, GO, PSS, and HEP. Such a coating also offers anti-fouling and anti-clotting capability. The resultant nanohybrids composed of conductive polymers (PEDOT), 2D materials (GO), and biopolymers (HEP) show excellent hemo- and bio-compatibility and would be applicable to improving cardiovascular stents.

## 2. Materials and Methods

### 2.1. Preparation of Electrochemical Polymerization

The SUS316L plate substrate (Sinkang Industries Co., Ltd., New Taipei City, Taiwan) was cut into 10 × 10 mm^2^ samples with thickness of about 0.5 mm. The plate was cleaned consecutively with detergent, ethanol, and deionized water and dried in an oven. The reacting solution was prepared by mixing EDOT (56.8 μL, Sigma, St. Louis, MO, USA) with PSS (142μL, Sigma, St. Louis, MO, USA) or GO (0.01 g, Angstron Materials, Taipei City, Taiwan) or HEP (0.02 g, Sigma, St. Louis, MO, USA) in 20 mL of deionized water under sonication for 3 h. The solution was placed into a three-electrode electrochemical analyzer (PGSTAT12, Metrohm Autolab B.V., Utrecht, Netherlands). The working electrode was the SUS316L plate, the counter electrode was a Pt wire, and the reference electrode was an AgCl electrode. A constant potential of 1 V (vs. Ag/Ag^+^) was used to produce PEDOT layers with a range of deposition densities (10–20 mC·cm^−2^).

### 2.2. Surface Characterization

The surfaces of bare SUS316L substrate and PEDOT composite films were examined using a field emission scanning electron microscope (FESEM, JSM6700F, JEOL, Japan), transmission electron microscope (TEM, JEOL JEM-2100), atomic force microscope (AFM, AFM, Dimension Edge, Bruker, America) operated in tapping mode, and X-ray photoelectron spectroscope (XPS, Thermo Scientific, Theta Probe, USA). The water contact angle was measured using a contact angle goniometer (DSA 100, Krüss GmbH, Hamburg, Germany) at room temperature. The contact angle was taken as an average of three repetitions.

### 2.3. Protein Adsorption

The plate was incubated in phosphate-buffered saline (PBS) containing 5 mg/mL of human serum albumin (HSA) in a 12-well tissue culture plate at 37 °C for 1 h. After that, the plate was washed three times using PBS in the 12-well tissue culture plate. Then, the plate was incubated in a 1 wt % aqueous solution of sodium dodecyl sulfate (SDS) before centrifuging in an incubator for 1 min to remove the proteins adsorbed on the plate. Finally, a bicinchoninic acid (BCA) kit was used to determine the HSA concentration in the SDS solution from the absorbance at 562 nm using a UV–vis spectrometer.

### 2.4. Blood Clotting Time (Activated Partial Thromboplastin Time, APTT)

Platelet-poor plasma (PPP) was obtained from Taipei Blood Center. The plates were incubated with 1 ml PPP at 37 °C for 1 h. Then, 50 μL of the PPP was added into the tube followed by adding 50 μL APTT reagent; the mixture was left to stand for 3 min, and 50 μL of CaCl_2_ solution was added. The APTT was determined using a coagulation analyzer (CA-50, Sysmex, Kobe, Japan).

### 2.5. Platelet Adhesion

Platelet-rich plasma (PRP) was obtained from Taipei Blood Center. The plates were incubated with PRP (10^8^ platelets/mL) at 37 °C for 1 h. Then the plates were rinsed with PBS before fixing the adhered platelets by immersing in glutaraldehyde. The number of platelets adhered on the plates was examined using SEM.

### 2.6. Biocompatibility Test

The cytotoxicity was evaluated with 3T3 fibroblasts. The culture medium was DMEM containing 10% fetal bovine serum (FBS) and 1% penicillin antibiotic (PNC). The SUS316L samples (1 cm × 1 cm) were autoclaved before being placed in the wells of a 12-well plate. In each well, 1 mL of the medium containing 10^5^ cells was added. Then, the plates were cultured in a humidified 37 °C, 5% CO_2_ incubator. On the first, third, and fifth days, the cell growth was determined by thiazolyl blue tetrazolium bromide (MTT) assay. Briefly, 20 μL of reagent was added into each well and incubated for 4 h at 37 °C. Then, dimethyl sulfoxide (DMSO) was mixed with the medium to dissolve the purple product. Finally, the absorbance at 570 nm was registered. The relative growth ratio (RGR) was calculated as follows:

$$\mathrm{RGR}=\frac{A_{570}\;\mathrm{at}\;\mathrm{time}\;t}{A_{570}\;\mathrm{at}\;\mathrm{time}\;0}$$

where A_570_ is the absorbance at 570 nm.

## 3. Results and Discussion

### 3.1. Surface Characterization

**Figure 1:**
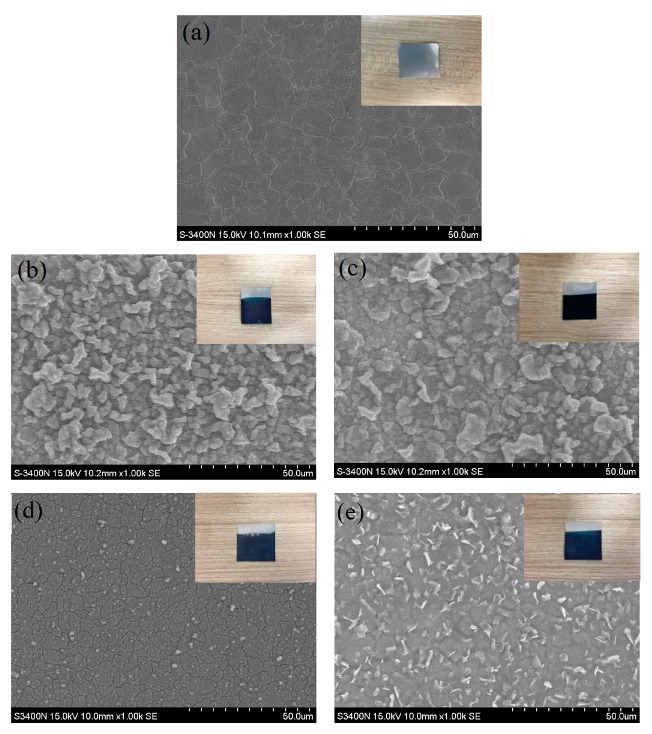
SEM images of (**a**) SUS316L stainless steel, (**b**) poly(3,4-ethylenedioxythiophene) (PEDOT)/heparin (HEP), (**c**) PEDOT/polystyrene sulfonate (PSS), (**d**) PEDOT/graphene oxide (GO), and (**e**) PEDOT/GO/HEP.

Figure 1 shows the surface morphology of bare and coated SUS316L plates. Macroscopically, these plates were similar in morphological features, except for the bare plate (Figure 1a). From the microscopic perspective, the SEM images (Figure 1b–e) revealed that PEDOT polymer composites were indeed deposited on the SUS316L plates. The surfaces of these coated plates were rough, primarily because of variation in the deposition rate or differences in substrate thickness, which induced uneven plating. The surface roughness of the thin films deposited in this study varied considerably because the electrochemical polymerization processes deposited thin films at a fast rate.

**Figure 2:**
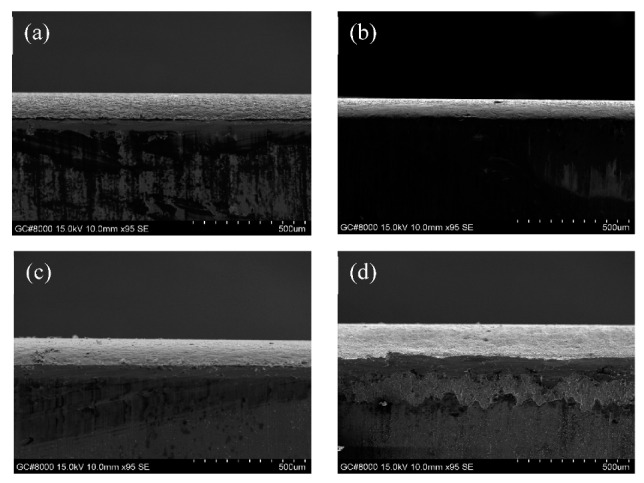
SEM images of the cross sections of (**a**) PEDOT/HEP, (**b**) PEDOT/PSS, (**c**) PEDOT/GO, and (**d**) PEDOT/GO/HEP.

Figure 2 shows a cross section of the thin-film coatings on SUS316L plates. The thickness of the films after electrochemical polymerization ranged between 80 and 146 μm, with PEDOT/PSS (80 μm) and PEDOT/GO/HEP (146 μm) having the smallest and largest thicknesses, respectively. Although doping with different polymers can control the film thickness, excessively thick films may crack easily. The films prepared in this study were uniform in thickness, and the film thicknesses would not affect their anti-clotting and anti-fouling abilities.

**Figure 3:**
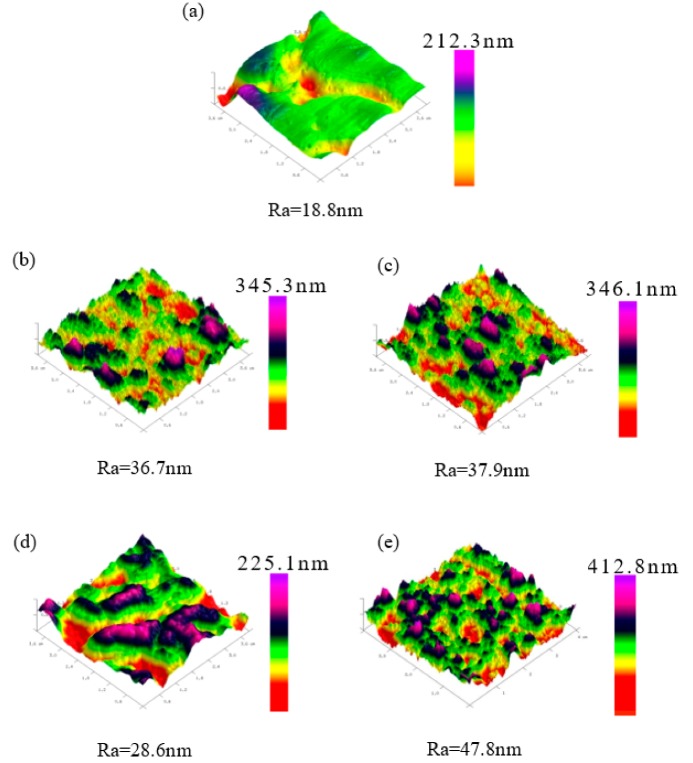
AFM images of (**a**) SUS316L, (**b**) PEDOT/HE, (**c**) PEDOT/PS, (**d**) PEDOT/GO, and (**e**) PEDOT/GO/HEP.

Figure 3 displays the AFM images of bare and thin-film-coated SUS316L plates. Comparing the surface roughness before and after electrochemical polymerization, the surface became rougher after electrochemical polymerization, indicating that the thin films were polymerized on the SUS316L plates. These films exhibited anti-fouling ability because PSS, heparin, and GO are negatively charged, and HSA is also negatively charged; thus, the anti-fouling abilities of these thin films were achieved through the repulsive interaction of negatively charged substances.

### 3.2. XPS Analysis

**Figure 4:**
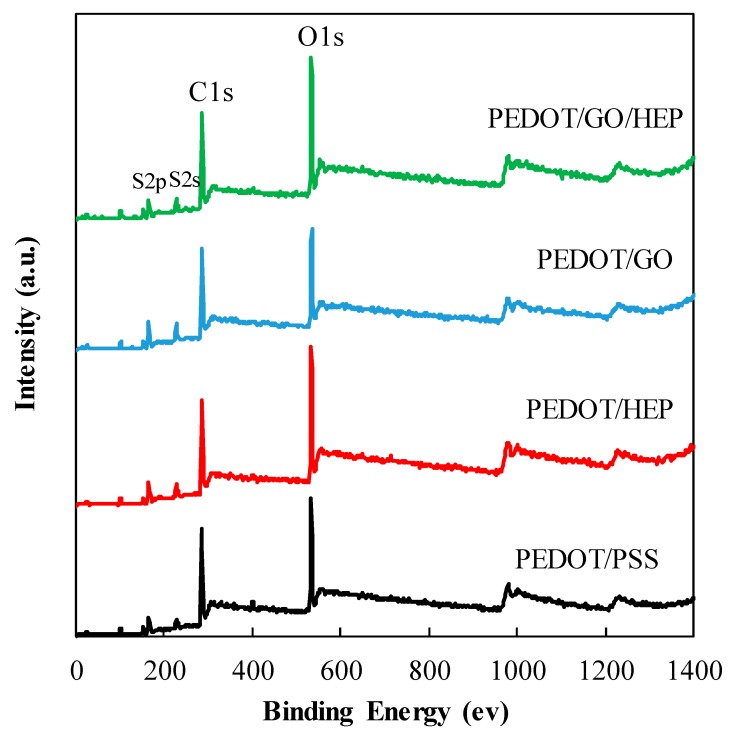
XPS full spectra of PEDOT/PSS, PEDOT/HEP, PEDT/GO, and PEDOT/GO/HEP.

The chemical bonding of various PEDOT composite thin films was verified using X-ray photoelectron spectroscopy (XPS). The full spectra (Figure 4) show that the thin film primarily contained S, C, and O. The ratio of S, C, and O changes with GO, PSS, and HEP addition. Compared with the PSS-doped film, the O1s peak of the GO-doped PEDOT thin film was slightly higher, owing to abundant oxygen-containing functional groups in GO. The S2p and S2s peaks at 166.5 and 220.5 eV are attributable to the sulfur polymers in PEDOT. Therefore, all the thin films were discovered to contain sulfur bonds. Finally, in the full spectrum of the doped HEP, the O1s peak was located at a higher energy than in that of GO. This was because HEP possesses more oxygen-containing functional groups than GO.

**Figure 5:**
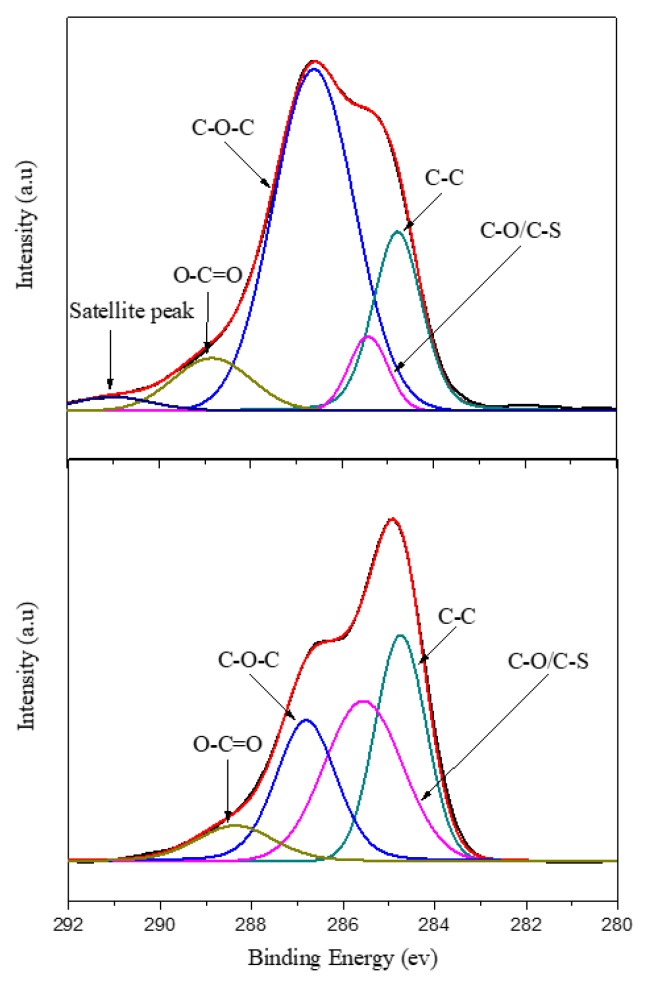
C1s energy spectra of PEDOT/GO and PEDOT/PSS.

Figure 5 displays the C1s spectra of the PEDOT/GO and PEDOT/PSS thin films. The peak at 286.8 eV denotes C–O–C bonds. PEDOT/GO was discovered to contain numerous oxygen-containing functional groups because of the presence of GO. These functional groups and the carbons in the EDOT monomer formed C–O–C bonds. Therefore, the area under the curve corresponding to C–O–C bonds is larger for PEDOT/GO than for PEDOT/PSS. Conversely, the area under the curve corresponding to C–S bonds is larger for PEDOT/PSS because both EDOT and PSS contain sulfur. These results are consistent with those reported in the literature.

**Figure 6:**
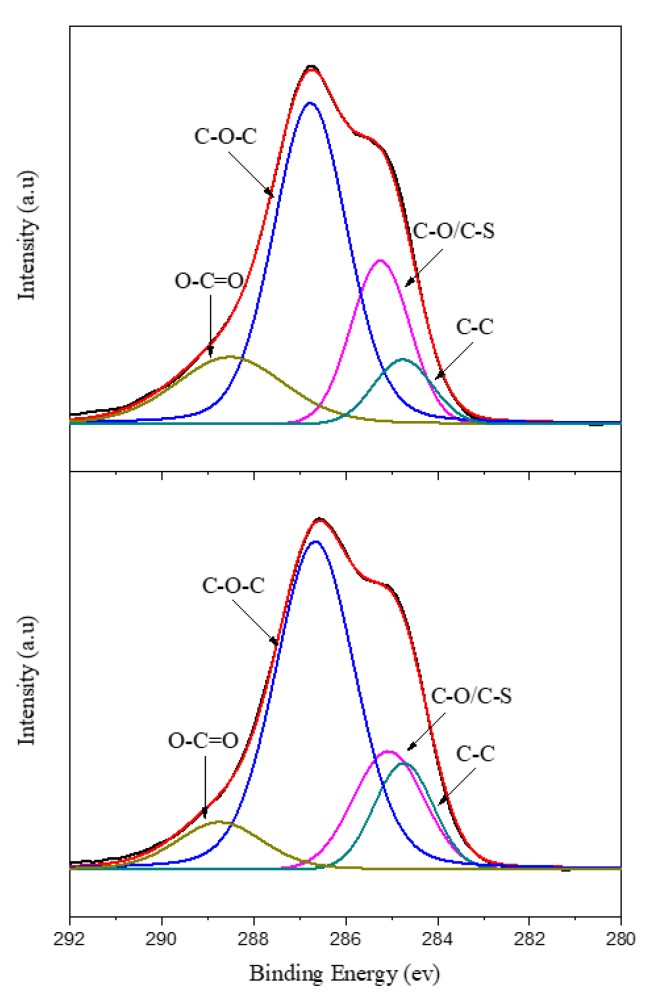
C1s energy spectra of PEDOT/HEP and PEDOT/GO/HEP.

**Figure 7:**
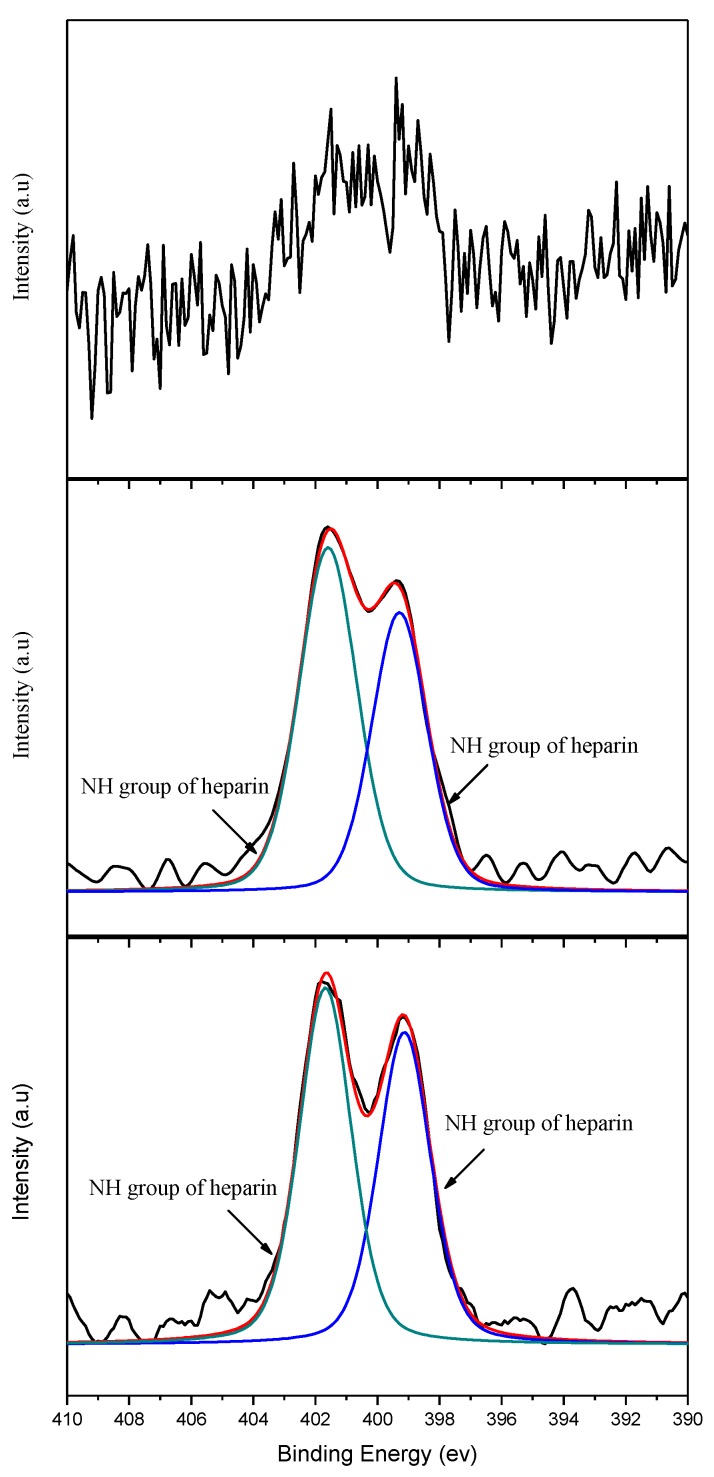
N1s energy spectra of PEDOT/GO, PEDOT/HEP, and PEDOT/GO/HEP.

Figure 6 shows the C1s energy spectra of PEDOT/HEP and PEDOT/GO/HEP. The areas under the curves corresponding to C–O–C and C–O/C–S for PEDOT/GO/HEP were nearly equal to those for PEDOT/HEP. However, the films could be distinguished based on the N1s spectra in Figure 7 because GO did not have a characteristic N1s peak, but HEP did. Therefore, the successfulness of the polymerization of PEDOT/HEP, PEDOT/GO, and PEDOT/GO/HEP can be verified by comparing the C1s and N1s spectra.

### 3.3. Water Contact Angle and Adhesion

**Figure 8:**
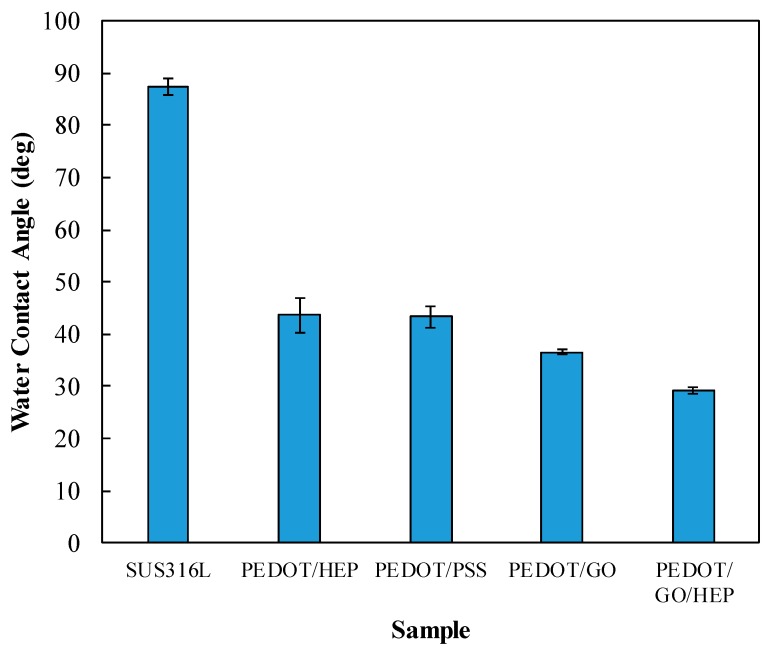
Water contact angles of SUS316L, PEDOT/GO, PEDOT/PSS, PEDOT/HEP, and PEDOT/GO/HEP.

As shown in Figure 8, the water contact angle of SUS316L was around 90°, indicating that this material was hydrophobic. On the other hand, the angles for all these PEDOT composite thin films were below 45°; thus, they were hydrophilic. The portion of dopant affected the water contact angle. The varying water angle can be attributed to the hydrophobicity of the polymer in the coating process. PEDOT/GO and PEDOT/PSS were found to have similar water contact angles. The HEP-doped film also exhibited a low water contact angle. Finally, when GO and HEP were doped in the thin films, the water contact angle was reduced to only 30°. This indicates that the coating caused a change in the water contact angle. Lower water contact angle means higher surface energy, and it is thus easier to adsorb HSA proteins on the film surface. However, the protein adsorption would also be influenced by the surface roughness and surface charge. In our case, we speculate that the surface charge will dominate the protein adsorption and platelet adhesion. This is discussed in Section 3.4.

**Figure 9:**
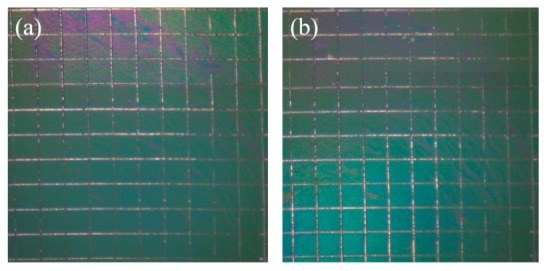
Photographs of the grid area of PEDOT/GO/HEP-coated SUS316L stainless steel before (**a**) and after (**b**) the cross-cut tape testing method.

According to the American standard test methods (ASTM), the adhesion between PEDOT/GO/HEP coating and SUS316L stainless steel was examined by a cross-cut tape testing method. The coated SUS316L stainless steels were crisscrossed to form a grid of 100 small squares to promote the feasible breakdown of the adhesion of films. Photographs of the PEDOT/GO/HEP-coated SUS316L stainless steel before and after the cross-cut tape testing method are shown in Figure 9. The grid of 100 small squares almost remained intact (damage did not exceed 5%). Strong adhesion strength (4B) was observed between the PEDOT/GO/HEP layers and SUS316L stainless steel due to the novel self-assembly construction by electrochemical polymerization from EDOT monomers with other derivatives.

### 3.4. Hemocompatibility

**Figure 10:**
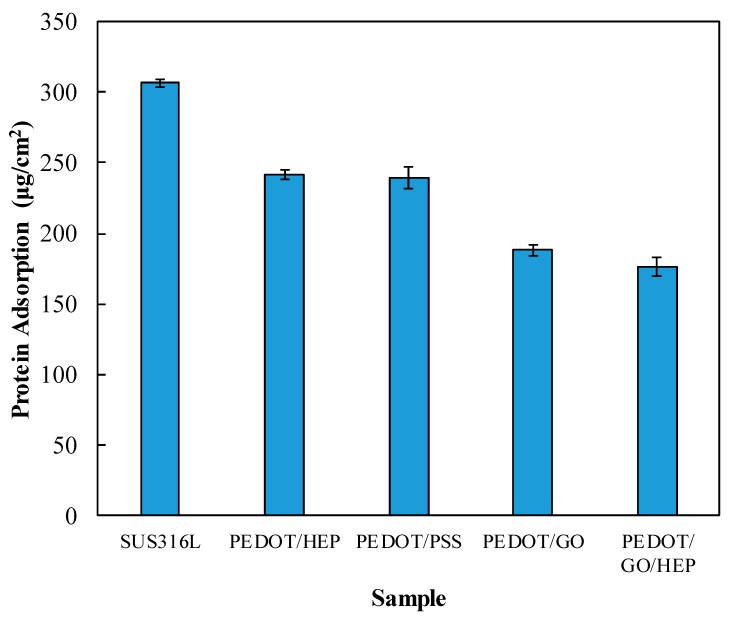
Protein adsorption on SUS316L, PEDOT/PSS, PEDOT/HEP, PEDOT/GO, and PEDOT/GO/HEP.

The hemocompatibility of the PEDOT composite film was evaluated based on three measures: HSA adsorption, platelet adhesion, and APTT. Figure 10 shows that the adsorbed amount of HSA on bare SUS316L substrate was higher than those with various PEDOT composite films. Both films doped with PSS and HEP reduced the adsorption by about 20%. A further reduction, by 40%, was observed for the GO-doped film. The thin film doped with both HEP and GO exhibited the least adsorption of HSA (42% less than the bare substrate), suggesting that GO contributed most of the anti-adsorption effect while HEP played a minor role. The negative charge of GO, PSS, and HEP can inhibit protein adsorption (negative charge surface), even though it displays a more hydrophilic surface compared to pristine SUS316L.

**Figure 11:**
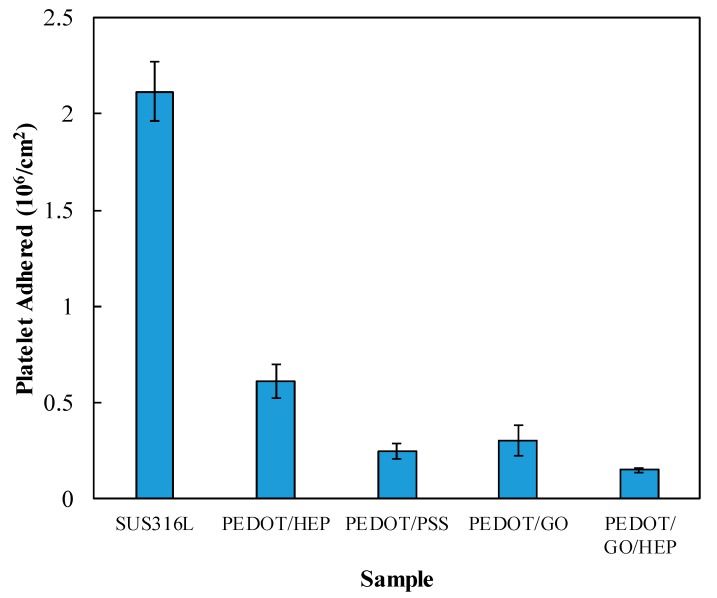
Platelet adhesion on SUS316L, PEDOT/PSS, PEDOT/HEP, PEDOT/GO, and PEDOT/GO/HEP.

Figure 11 shows the amounts of platelets adhered on bare and coated SUS316L plates. The surface density of platelets on bare SUS316L was much higher than those on the PEDOT thin films. In particular, PEDOT/GO/HEP exhibited the least adhesion of HSA—only 7% of the surface density of platelets on bare SUS316L. This confirmed that PEDOT/GO/HEP can suppress the adhesion of platelets.

**Figure 12:**
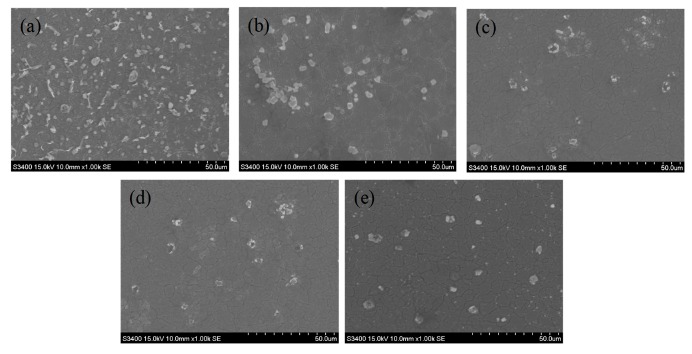
SEM images of human platelets adherent to (**a**) SUS316L, (**b**) PEDOT/HEP, (**c**) PEDOT/PSS, (**d**) PEDOT/GO, and (**e**) PEDOT/GO/HEP substrates.

**Figure 13:**
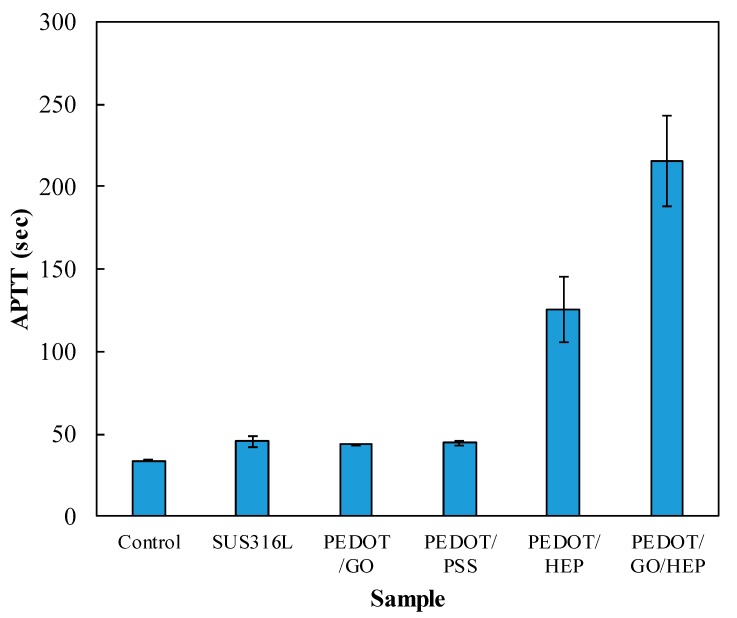
Coagulation times for the composite films.

The roughness of the sample increases when a sample is coated by a film, which induces more platelets to adhere on the surface. However, the platelet adhesion and the morphology would also be influenced by the surface energy and surface charge. In our study, we found that the effect of surface charge was higher than that of the surface roughness and surface energy because the negative charge of GO, PSS, and HEP repels the negative charge of the platelets. The adhesion and morphology of the platelets can be observed in Figure 12. Spread dendritic and spreading platelets were found in PEDOT/PSS (Figure 12c) and PEDOT/GO (Figure 12d). However, only round and dendritic platelets were observed in PEDOT/HEP (Figure 12b) and PEDOT/GO/HEP (Figure 12e). Thus, PEDOT/HEP and PEDOT/GO/HEP can prolong the blood coagulation time very well, as shown in Figure 13.

Figure 13 shows the APTT of the PPP contacting bare SUS316L and PEDOT composite films. The APTT of the control (healthy people) was 33.9 ± 0.4 s. The APTT values for SUS316L, PEDOT/GO, and PEDOT/PSS were 45.8 ± 3.2, 44.2 ± 0.2, and 44.8 ± 1.4, respectively. However, when HEP was doped, the APTT for PEDOT/HEP was 125 ± 20 s, more than 3 times that of the control. Further, the APTT for PEDOT/GO/HEP was 216 ± 27 s, possibly due to the multilayer structure of GO, wherein heparin could be trapped between layers and lead to a coagulation time 5 times longer than the control. This study thus demonstrated that by doping GO and HEP to PEDOT, the composite films exhibited much longer APTT and would thus greatly retard blood coagulation.

### 3.5. Cytotoxicity

The ultimate objective of this experiment was to apply this electrochemical polymerization approach to a cardiovascular stent implanted in the body. Biocompatibility is vital; poor biocompatibility in an implanted object may induce allergies and septicemia that can be fatal. The in vitro cytocompatibility of PEDOT composite films was evaluated based on the proliferation of NIH 3T3 cells, which are mouse embryonic fibroblasts. They have been widely used in biocompatibility tests.

**Figure 14:**
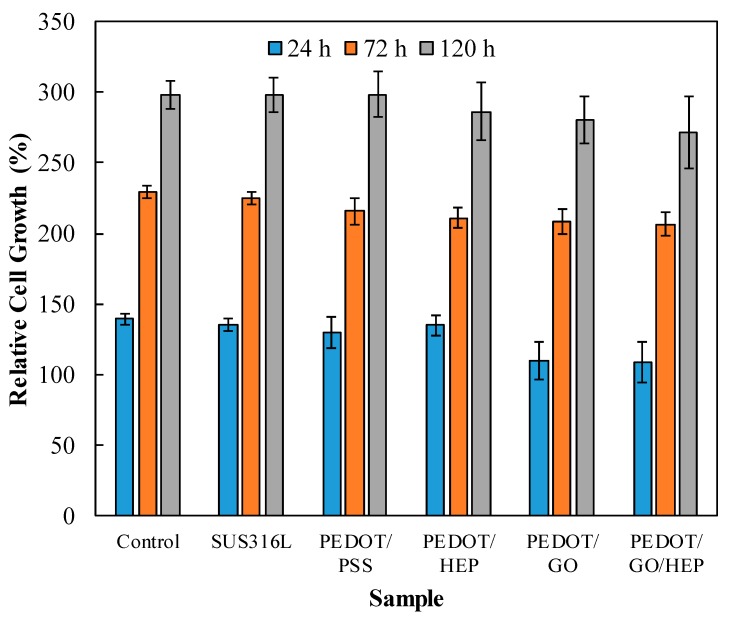
Biocompatibility of the composite films.

Figure 14 shows that 3T3 cells proliferated for all substrates on the first, third, and, fifth days. The results show that the RGR of the bare SUS316L was slightly lower than that of the control, although the difference was insignificant. The cells on the PSS- and HEP-doped PEDOT films exhibited RGR comparable to that of the control, whereas those on GO- and GO/HEP-doped PEDOT films were about 90% of that of the control. However, all these RGR values were above 100%, indicating that the PEDOT composite films exhibited no cytotoxicity to 3T3 fibroblasts. Thus, this electrochemical polymerization could be applicable to implantable biomedical devices such as cardiovascular stents.

## 4. Conclusions

In this paper, nanohybrid films of poly(3,4-ethylenedioxythiophene) (PEDOT) doped with GO, PSS, or HEP were successfully electrochemically polymerized on SUS316L substrates as indicated by the results of SEM, AFM, and XPS studies. These PEDOT composite films decreased the water contact angle by more than half compared to that of bare SUS316L, indicating an improvement in the wettability of the substrate and leading to less adsorption of HSA and platelets. These composite films also exhibited longer APTT. Among these substrates, the PEDOT/GO/HEP films exhibited the least HSA adsorption and platelet adhesion because the negative charge of GO produces an expelling force against negatively charged proteins and platelets. Furthermore, these films showed no cytotoxicity to 3T3 fibroblasts. Therefore, electrochemically polymerized PEDOT nanohybrids are potentially applicable in biomedical coatings to improve the hemocompatibility of blood-contacting devices such as cardiovascular stents.
